# Correlations of Metabolic Components with Prostate Volume in Middle-Aged Men Receiving Health Check-Up

**DOI:** 10.1371/journal.pone.0145050

**Published:** 2016-01-05

**Authors:** Teng-Kai Yang, Peter Woo, Hung-Ju Yang, Hong-Chiang Chang, Ju-Ton Hsieh, Kuo-How Huang

**Affiliations:** 1 Department of Surgery, Yonghe Cardinal Tien Hospital, New Taipei City, Taiwan; 2 College of Medicine, Fu Jen Catholic University, New Taipei City, Taiwan; 3 Department of Internal Medicine, Yonghe Cardinal Tien Hospital, New Taipei City, Taiwan; 4 Department of Urology, College of Medicine, National Taiwan University and National Taiwan University Hospital, Taipei, Taiwan; The Chinese University of Hong Kong, HONG KONG

## Abstract

**Objectives:**

To investigate the impact of metabolic components and body composition indices on prostate volume (PV) in a population of middle-aged men receiving health check-ups.

**Methods:**

Six hundred and sixteen men receiving health assessments were stratified to large and small prostates based on the cut-off of median PV. Their demographic data, health history, and international prostate symptoms scores (IPSS) were collected. Metabolic components and body composition indices were compared between subjects with large and small prostates. Moreover, the correlations between these parameters and PV were analyzed by multivariate logistic regression.

**Results:**

The median PV was 27 mL and mean age was 54.8 years. Subjects with large PV were older (56.5 vs. 52.7 years) and had higher serum prostate specific antigen (PSA) level (1.73 vs. 0.96 ng/mL), higher IPSS score (8.37 vs. 6.16), and higher body fat, body mass, and waist circumference (all *p*<0.05). In multivariate analysis, age (OR, 2.45; 95%CI, 1.74–3.45), serum PSA (OR, 2.75; 95%CI, 1.96–3.86), waist circumference (OR, 1.45; 95%CI, 1.02–2.07), fatness (OR, 1.47; 95%CI, 1.04–2.09), and body fat mass (OR, 1.43; 95%CI, 1.00–2.03) were significantly correlated with PV of study subjects. In subgroup analysis, raised waist circumference (OR, 1.89; 95%CI, 1.00–3.59) was the independent predictor of PV in subjects with bothersome lower urinary tract symptoms.

**Conclusions:**

Several metabolic components and body composition indices are significantly associated with PV of middle-aged men, including raised waist circumference, fatness, and body fat mass. Raised waist circumference is the only independent predictor of PV in middle-aged men with bothersome LUTS.

## Introduction

Benign prostatic enlargement is one of the most common diagnoses for middle-aged men with lower urinary tract symptoms (LUTS).[1, 2] Prostate volume (PV) measurement becomes increasingly important because of the connections to disease progression, the need for surgery, treatment response prediction and therapeutic options.[3, 4] Though aging and androgen effect have been established as etiologies of benign prostate enlargement, other determinants has not been well explored especially in middle-aged men with the presence of LUTS.[5]

Metabolic syndrome (MetS), a cluster of metabolic disorders, was characterized by insulin resistance with compensatory hyper-insulinemia and encompasses components of central obesity, arterial hypertension, dyslipidemia, and glucose intolerance. MetS makes tremendous impact on public health and social economy.[6] Emerging evidence has indicated the connections of MetS and benign prostate enlargement.[5, 7] Recently, many parameter for the measurement of metabolic components have been developed. Among them, obesity indices such as increased body mass index, fat mass and body fat percentage has been reported to be remarkably associated with benign prostate enlargement.[8, 9] Moreover, body composition indices, including lean mass, fat mass and total body water were thought to be good predictors for obesity-related diseases as well.[10–12] In addition to the components of MetS, these well-developed body composition indices may potentially serve as the critical factors to predict PV.

This prospective study investigated the correlations of PV with MetS, metabolic components and body composition indices in middle-aged men receiving health check-up.

## Materials and Methods

### Ethics statement

All the written informed consents from the patients were obtained. The study was approved by the institutional review board at National Taiwan University Hospital (No: 201407108RINC) and was performed in accordance with the Helsinki Declaration.

### Study subjects

Six hundred and sixteen males aged ≥40 years were recruited from a self-paid medical check-up at the Health Management Center in National Taiwan University Hospital formed the study sample. Prior to examination, a self-administered questionnaire was used to collect information on participant demographic characteristics, clinical symptoms, and medical and medication histories. The exclusion criteria were as follows: 1) history of prostate cancer; 2) history of prostate surgery, including prostate biopsy within 4 weeks; 3) use of hormone replacement therapy or 5-alpha-reductase inhibitor. We assumed that these factors potentially interfered with major parameters measurement, including prostate volume, International Prostatic Symptoms Score (IPSS) and serum prostate specific antigen (PSA).

All of the study subjects completed a validated Chinese version of IPSS questionnaire and received urinary flowmetry assessment. Prostate volume was measured by digital rectal examination by an experienced urologist using the GE Healthcare LOGIQ P5 trans-rectal ultrasound system. The area of greatest transverse diameter in the transverse view, antero-posterior, and transverse dimensions of the prostate were measured. Sagittal scanning was then performed and the distance from the base to the apex in the median plane was measured along the longitudinal dimension. Mets was defined according to the recent consensus report of the National Cholesterol Education Program’s Adult Treatment Panel III,[13] which were applied to ascertain that the MetS cases had a major component of central obesity defined by waist circumference greater than 90 cm in an Asian male, and two of other components as follows: 1) increased blood pressure (at least 130/85 mmHg or undergoing medication for hypertension); 2) increased fasting blood sugar (≧5.5 mmol/L or 100 mg/dL); 3) increased serum triglyceride (≧1.7 mmol/L or 150 mg/dL); 4) reduced HDL cholesterol (≦1.0 mmol/L or 40 mg/dL). Additionally, obesity was defined by BMI≧25 according to WHO guideline for Asian male.[14]

As for body composition indices, bioelectric impedance analysis was utilized and fat body mass was obtained by subtracting lean body mass from body weight. Percent body fat was calculated from the ratio of body fat mass versus body weight. The total body water was calculated from the impedance analysis, while the lean body mass was calculated as TBW/0.72.[15–17] Body protein, muscle, mineral, and extracellular and intracellular fluid were calculated accordingly.

### Statistical analysis

Continuous variables were analyzed with the Student *t* test and are presented as mean ± standard deviation (SD). Categorical variables were analyzed by the chi-square test and recorded as frequency or percentage. The two-sided alpha level was 0.05 and statistical significance was set at *p*<0.05. In order to examine the impact of potential predictors on PV, we analyze the data with logistic regression model, which continuous variables including age, serum PSA and body composition indices, were transfer to binary outcomes utilizing median as a cut-off point. Due to the similarities between metabolic components and body composition indices, collinearity was evaluated as well. All statistical analysis was done using the commercial statistical software (SPSS version 13.0 for Windows, SPSS Inc., Chicago, IL).

## Results

The clinical and demographic characteristics of the study subjects were stratified to large versus small prostate based on the cut-off of median PV (27 mL). As shown in Table 1, the mean PV, small vs. large prostate was 21.1±3.79 vs. 37.5±11.1 mL (*p*<0.001). The mean age of subjects with small and large PV was 52.7 and 56.5 years, respectively (*p*<0.001). Similarly, serum PSA level was significantly higher in subjects with large prostate compared to those with small prostate (1.73±1.80 vs. 0.96±0.86 ng/ml, *p*<0.001).

**Table 1:** Demographic characteristics of study subjects stratified by small and large prostate volume (cutoff by median prostate volume, 27 mL).

	Small prostate(n = 298)	Large prostate(n = 318)	*p*
**Age (yrs)**	52.7±6.59	56.5±6.47	<0.001
**Body Height (cm)**	170±5.86	170±6.26	0.72
**Body weight (kg)**	71.5±9.38	72.5±9.67	0.19
**BMI (kg/m**^**2**^**)**	24.8±2.75	25.2±2.91	0.95
**Prostate volume (ml)**	21.1±3.79	37.5±11.1	<0.001
**Serum PSA (ng/dl)**	0.96±0.86	1.73±1.80	<0.001
**IPSS**			
**Total**	6.16±5.41	8.37±6.81	<0.001
**QoL score**	2.15±1.27	2.41±1.33	0.012
**Storage score**	2.77±2.15	3.70±2.76	<0.001
**Frequency**	1.38±1.32	1.65±1.43	0.016
**Urgency**	0.45±0.75	0.81±1.14	<0.001
**Nocturia**	0.94±0.80	1.24±0.97	<0.001
**Voiding score**	3.39±3.94	4.67±4.68	<0.001
**Incomplete emptying**	1.12±1.35	1.46±1.51	0.003
**Intermittency**	0.85±1.24	1.19±1.43	0.002
**Weak stream**	0.93±1.45	1.30±1.63	0.003
**Straining**	0.49±0.94	0.72±1.16	0.008
**IPSS grade**			0.001
**Mild**	210 (70.5)	181 (56.9)	
**Moderate**	79 (26.5)	113 (35.5)	
**Severe**	9 (3.0)	24 (7.5)	
**Qmax <15 ml/sec**	80 (26.8)	102 (32.1)	0.16

Numeric data is expressed as mean±standard deviation (SD) and categorical data as a number (percentage).

Within the whole study sample, 225 (36.5%) had bothersome LUTS (IPSS ≥8). Subjects with large prostate suffered from significantly higher IPSS score, storage score, voiding score, and the score of each IPSS item compared to those with small prostate (all *p*<0.05).

Table 2 demonstrates the body composition indices and metabolic components in subjects with small and large PV. We observed subjects with large PV had significantly higher fatness, body fat percentage, body fat mass, and increased waist circumference compared to those with small PV (all *p*<0.05) (Table 2). In contrast, protein, muscle and mineral compositions were comparable between the two groups.

**Table 2:** Body composition and metabolic syndrome parameters in study subjects with small vs. large prostate.

	Small prostate (n = 298)	Large prostate (n = 318)	*p*
**Body composition**			
**Fatness (kg)**	9.93±13.0	12.1±13.6	**0.048**
**Body fat percentage (%)**	25.0±4.40	25.8±4.40	**0.028**
**Body fat mass (kg)**	18.2±5.11	19.0±5.32	**0.05**
**Lean body mass(kg)**	53.3±5.20	53.5±5.41	0.61
**Total body water (kg)**	38.4±3.75	38.4±4.34	0.87
**Basal metabolic rate(kcal/day)**	1557±117	1567±123	0.34
**Intracellular Fluid (kg)**	25.6±2.50	25.7±2.59	0.61
**Extracellular Fluid (kg)**	12.8±1.25	12.8±1.48	0.90
**Protein (kg)**	10.8±1.18	10.8±1.21	0.85
**Muscle (kg)**	49.0±5.51	49.2±5.64	0.65
**Mineral (kg)**	4.09±0.57	4.15±0.59	0.25
**Metabolic syndrome**	83 (27.9)	92 (28.9)	0.77
**raised WC**	136 (45.6)	173 (54.4)	**0.03**
**raised BP**	124 (41.6)	153 (48.1)	0.10
**raised FBS**	75 (25.2)	80 (25.2)	0.99
**raised TG**	86 (28.9)	89 (28.0)	0.81
**reduced HDL**	106 (35.6)	96 (30.2)	0.16
**Obese (raised BMI)**	130 (43.6)	153 (48.3)	0.25

Numeric data is expressed as mean±SD and categorical data as a number (percentage).

Abbreviations: WC, waist circumference; BP, blood pressure; FBS, fasting blood sugar; TG, serum triglyceride; HDL, high-density lipoprotein; BMI: body mass index

We then analyzed the potential predictors of PV by logistic regression model. As shown in Table 3, age and serum PSA were analyzed as categorical variables by the cut-off median value. Body composition indices were checked but excluded from the model in the stepwise selection due to marked collinearity and model simplification.

**Table 3:** Odds ratios of covariates, large vs. small prostate volume, analyzed by logistic regression model in whole study sample and subjects subcategorized by the presence of bothersome LUTS.

	Total	No bothersome LUTS	Bothersome LUTS
	(n = 616)	(n = 391)	(n = 225)
**Age**	2.45 (1.74–3.45) *	1.93 (1.26–2.97) *	3.29 (1.77–6.09) *
**Serum PSA**	2.75 (1.96–3.86) *	2.32 (1.53–3.52) *	4.61 (2.43–8.74) *
**Raised WC**	1.45 (1.02–2.07) *	1.20 (0.77–1.88)	1.89 (1.00–3.59) *
**Raised BP**	1.25 (0.88–1.78)	1.03 (0.66–1.61)	1.88 (1.01–3.52) *
**Raised FBS**	0.84 (0.56–1.26)	1.10 (0.67–1.80)	0.54 (0.26–1.11)
**Raised TG**	1.06 (0.71–1.57)	0.96 (0.60–1.54)	1.64 (0.72–3.70)
**Reduced HDL**	0.69 (0.47–1.01)	0.77 (0.48–1.21)	0.58 (0.29–1.16)

*: statistically significant

Odds Ratio: large vs. small prostate volume

Of the whole study sample, age (OR, 2.45; 95%CI, 1.74–3.45), serum PSA (OR, 2.75; 95%CI, 1.96–3.86), and raised waist circumference (OR, 1.45; 95%CI, 1.02–2.07) were independent predictors of PV. The subgroup analysis for subjects with bothersome LUTS, we found age, serum PSA, raised waist circumference, and increased blood pressure were independent predictors of PV (OR, 3.29, 4.61, 1.89 and 1.88, respectively; all *p*<0.05). For subjects without bothersome LUTS, age and serum PSA were the two independent predictors of PV.

As is shown in Table 4, we examined whether obesity indices were significantly independent predictors of bothersome LUTS after adjustment of established predictors such as age, PSA as well as MetS components such as higher blood pressure, raised fasting blood sugar, increased triglyceride, and reduced high density lipoprotein.[5, 7, 9] Increased waist circumference, obese (raised body mass index), fatness, body fat percentage, and body fat mass were subsequently selected for further analysis due to relatively small p value in the univariate analysis. The results showed there was no significant collinearity among the variables. After adjustment, increased waist circumference, fatness, and body fat mass were independent predictors for PV in all subjects (all *p*< 0.05); while increased waist circumference was the only independent predictor in subjects with bothersome LUTS.

**Table 4:** Adjusted odds ratio^**^ to measure the impacts of metabolic components and body composition indices on PV in whole study sample and subjects subcategorized by the presence of bothersome LUTS.

Variable	Total	Men without bothersome LUTS	Men with bothersome LUTS
	(n = 616)	(n = 391)	(n = 225)
**Raised WC**	1.45 (1.02–2.07) *	1.20 (0.77–1.88)	1.89 (1.00–3.59) *
**Obese (raised BMI)**	1.42 (0.99–2.02)	1.45 (0.93–2.26)	1.40 (0.75–2.62)
**Fatness**	1.47 (1.04–2.09) *	1.47 (0.95–2.26)	1.49 (0.79–2.79)
**Body fat percentage**	1.36 (0.96–1.92)	1.27 (0.82–1.96)	1.41 (0.76–2.61)
**Body fat mass**	1.43 (1.00–2.03) *	1.46 (0.94–2.25)	1.43 (0.75–2.71)

* statistically significant

**After adjustments for age, serum PSA, increased BP, higher FBS, raised TG, and reduced HDL.

Odds Ratio: large vs. small prostate volume

## Discussion

Prostate enlargement is considered to be strongly correlated with LUTS. Recently, MetS has been reported to be associated with reduced LUTS in middle-aged Asian men.[18, 19] This can be explained by the favorable effect for early offstage hyper-insulinemia. Nevertheless, the exact correlation between MetS and PV has not been well established, especially in middle-aged men. In the present study, increased waist circumference (central obesity) is confirmed to be a significantly predictor of PV in middle-aged men, especially those with bothersome LUTS.

Increased sympathetic nervous system activity and obesity-related inflammation are two main effects of Insulin resistance with secondary hyper-insulinemia. These factors have been associated with prostate growth and enlargement [20–24] and may play a major role in the early stage of prostate growth for men with MetS. Some researches even advocate the use of anti-inflammatory medications for the treatment of early stage BPE. Some studies indicate that obese children have a significant increase in the concentrations of inflammatory markers such as hsCRP and IL-6. These studies imply that a pro-inflammatory status in pre-clinical MetS may be correlated with benign prostate enlargement.[25]

Obesity may also be correlated with prostate enlargement in relatively younger adults.[26] Nevertheless, which obesity index can predict prostate volume in middle-aged men still remains controversial. Yang et al. report that waist circumference is actually well correlated with prostate volume instead of body mass index or waist-to-hip ratio in men aged between 40 and 69 years.[27] In the present study, waist circumference, instead of body mass index, is significantly associated with PV.

Body composition indices has been associated with prostate cancer.[28] Fowke et al. indicate that high grade prostate cancer is strongly associated with percent body fat and body fat mass, which reflects shared genetic or hormonal factors for both adiposity preservation and prostate carcinogenesis. In the present study, obesity indices, including fatness and body fat mass, are two independent predictors of PV and the genetic, hormonal, or nutritional factors may account for the connection between body adiposity and benign prostate enlargement.

To date, this is the first study to evaluate the correlation of PV with metabolic components and body composition indices. The results show that increased waist circumference and blood pressure are two independent predictors for middle-aged men with bothersome LUTS and body component indices, including fatness and body fat mass, are better predictors of PV compared to body mass index.

Nonetheless, this study has several limitations that must be considered when interpreting the results. First, PV measurement by trans-rectal ultrasound may not represent the actual prostate volume. There are inter-observer variations and controversies in prostate volume estimation by ultrasonography.[29] One study reports that the most accurate method for prostate volume estimation varies with different volume ranges.[30] Second, subjects in present study were relatively healthy subjects undergoing medical check-up, which may be biased the results due to the healthy worker effect. We should be cautious of over-generalizing the study conclusions to the general population. Further studies are warranted to extrapolate these findings to the general male population. Lastly, it is impossible to completely exclude all patient with prostate cancer without surgical resection or random biopsy of the prostate. Thus, the results should be used with caution, unless prostate cancer has been sufficiently excluded in all patients.

## Conclusions

Our findings suggest several metabolic components and body composition indices are independent predictors of PV in middle-aged men, including raised waist circumference, fatness, and body fat mass, instead of the generally used obesity index, body mass index. Raised waist circumference is the only factor significantly associated with PV among study subjects with bothersome LUTS.
